# Securely Measuring the Overlap between Private Datasets with Cryptosets

**DOI:** 10.1371/journal.pone.0117898

**Published:** 2015-02-25

**Authors:** S. Joshua Swamidass, Matthew Matlock, Leon Rozenblit

**Affiliations:** 1 Department of Pathology, Washington University School of Medicine, St. Louis, MO, USA; 2 Prometheus Research LLC, New Haven, CT, USA; Children’s Medical Research Institute, AUSTRALIA

## Abstract

Many scientific questions are best approached by sharing data—collected by different groups or across large collaborative networks—into a combined analysis. Unfortunately, some of the most interesting and powerful datasets—like health records, genetic data, and drug discovery data—cannot be freely shared because they contain sensitive information. In many situations, knowing if private datasets overlap determines if it is worthwhile to navigate the institutional, ethical, and legal barriers that govern access to sensitive, private data. We report the first method of publicly measuring the overlap between private datasets that is secure under a malicious model without relying on private protocols or message passing. This method uses a publicly shareable summary of a dataset’s contents, its cryptoset, to estimate its overlap with other datasets. Cryptosets approach “information-theoretic” security, the strongest type of security possible in cryptography, which is not even crackable with infinite computing power. We empirically and theoretically assess both the accuracy of these estimates and the security of the approach, demonstrating that cryptosets are informative, with a stable accuracy, and secure.

## Introduction

Integrating and analyzing large amounts of data is proving to be a powerful method for exploring and understanding everything around us [1]. In an increasingly data-driven world, we have two contradictory impulses: both to *share* data and to keep it *private*. On one hand, data is powerful and sharing it lets us answer big questions that are unapproachable by other means. On the other hand, there is real danger in sharing it publicly or indiscriminately, so there are institutional barriers governing access to useful data.

Often, especially when human subjects are involved, the value of sharing data depends on the overlap between private datasets. The amount of overlap determines if there is reason to work through institutional, legal, or ethical barriers governing access to private data. Herein lies the problem; we often need to know the overlap between datasets to justify sharing. When trying to *link* entities between repositories (for example, to connect medical records in one database with genetic data in another database), we want there to be a high overlap. When trying to *extend* data by aggregating entities between several repositories (for example, to aggregate patients with a specific disease from two databases), we want there to be a low overlap. But how can we know the overlap between private datasets before sharing them?

### Existing approaches

Measuring the overlap, the intersection, between private datasets is a well-defined problem in cryptography [2]. Several solutions have been proposed, and they fall into three broad categories.

First, there are methods that share public IDs for each item in the dataset that retain a one-to-one (or nearly one-to-one) relationship to private IDs that need to be kept secret [3–6]. These methods attempt to secure themselves by using a one-way hash to generate public IDs, so it is non-trivial to recover private IDs from a list of public IDs. This strategy is inadequate; Attackers can use a “white-pages” attack, by maliciously enumerating private IDs, and testing them either in sequence or at once for membership in another private dataset [4, 7, 8]. Comprehensive attacks, which explore all possible private IDs, are also possible on some hash functions [9]. Countering these attacks requires keeping secret either the public IDs, the hash function, or a password, a solution may work in trusted environments (such as in a honest-but-curious model), but is inadequate for public, untrusted environments.

Second, there are methods that compute the exact overlap between private datasets without relying on shared public IDs [7, 10–21]. A full review is beyond the scope of this paper, but the general approach is to exploit the fact that it is easy to multiply large integers but it is computational difficult to factor the product of large integers [22, 23]. This mathematical fact enables elaborate, message-passing protocols for computing overlap, which rely on this computational barrier to ensure their security. Without getting into the technical details, it is possible to use any one of these strategies to compute the intersection between private datasets, while preserving the privacy of unmatched items in the datasets. However, all the reported methods in this class have two features that make them unsuitable for our purposes: (1) they reveal too much information about private datasets by enabling computation of the exact overlap and (2) they require complicated message-passing between parties to compute the overlap between datasets, without providing a publishing mechanism for set intersection computation. The first problem is most substantial because it is not easily fixed. All methods that compute overlap exactly are vulnerable to a white-pages attack in a malicious environment.

Third, there are methods that rely on a randomized data structure, a Bloom filter, to inexactly estimate the overlap between sets [24–28]. These methods work by creating a public summary of each dataset, a bit vector, also known as a Bloom filter or chemical fingerprint, from which overlap can be estimated. However, the problem with Bloom filters is two fold: (1) their accuracy rapidly degrades as the number of items in the private dataset grows [24] and (2) there is not yet a statistical framework for computing significance, power, confidence intervals, critical values, and information risk for overlap estimates using them. The first problem is the most substantial because it cannot be easily rectified. Bloom filter estimates of overlap are most accurate for small datasets, and become progressively less accurate as datasets grow in size. As items are added, they become saturated and less accurate. In many applications, this deficiency is typically addressed by increasing the length of the bit vector. But increasing the length will make the filters for small datasets much less secure.

### Proposed approach

**Fig 1:**
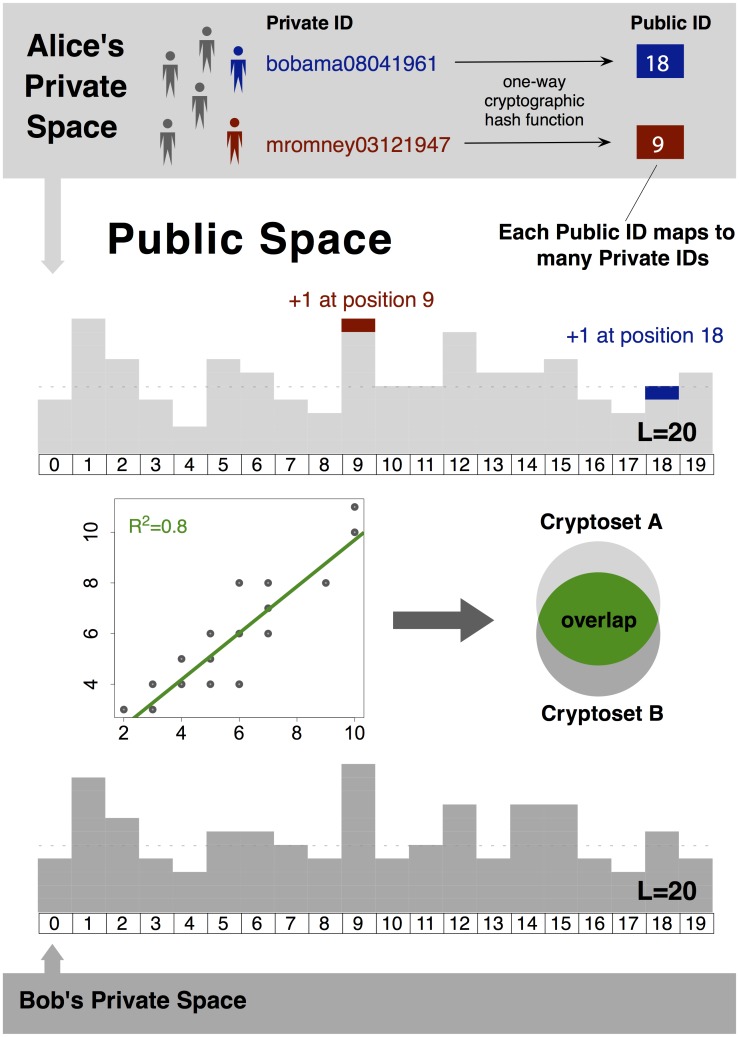
Cryptosets are shareable summaries of private data, from which estimates of overlap can be computed. They are constructed using a cryptographic hash function to transform private IDs from a dataset into a limited number of public IDs, and then combining these public IDs into a histogram. From this histogram (about 1000 IDs long in practice), the overlap between private datasets can be estimated in a public space. The security of cryptosets relies on the fact that several private IDs map to each public ID. The estimates are based on the Pearson correlation between cryptosets, and can only measure overlap at a predetermined resolution.

Here, we propose a new solution to the private set intersection problem (Fig. 1). Our approach, cryptosets, is an extension of Bloom filters that use counts at each vector element instead of bits: “count” Bloom filters or “count” chemical fingerprints [29–32]. Count filters have been proposed in the literature for the purpose of improving chemical similarity and supporting delete operations, but not for the purpose of securely computing overlap. This method uses a public algorithm to generate a public summary of any private dataset’s contents, which we call its cryptoset. The overlap between two private datasets can be estimated by comparing their cryptosets. At the same time, it is not possible to determine which items are in a private dataset from its cryptoset. Unlike other approaches to this problem [4, 8, 20], the item-level security arises from statistical properties of cryptosets rather than the secrecy of the algorithm or computation difficulty, so cryptosets can be shared in public, untrusted environments.

It is worth considering the properties of an ideal solution. First, the ideal solution would be *informative* about private datasets, enabling computation of their overlap. A noisy estimate of overlap is informative if it is accurate enough to inform good decisions about data sharing. Second, this solution’s accuracy would be *stable*, estimating accuracy at the same resolution for both small and large datasets with a tunable accuracy. Third, the solution would be *secure*, making it impossible to determine the membership of a specific entity in a private dataset. Critically, security dictates that overlap estimates cannot be exact, but must be intrinsically noisy. Finally, the solution would be *public*—neither relying on secret passwords, private hash functions, nor back-and-forth message passing—with summaries publishable in public spaces. These criteria immediately rule out all existing approaches and motivate the design of a better algorithm. In contrast, we will see that cryptosets satisfy all four of these properties; they are informative, stable, secure, and public.

Here, we develop a statistical framework for cryptosets from which overlap estimates can be computed, as well the confidence intervals, significance, and statistical power of these estimates. This framework makes clear that cryptoset accuracy is stable, and does not reduce as private datasets grow and their cryptosets saturate. Moreover, we prove that as size of private datasets increase, cryptosets approach information-theoretic security, the strongest type of security possible [33]. In contrast, we show that overlap estimates using Bloom filters—a related method in the literature—are unstable and less accurate than cryptoset estimates. Together these specific contributions provide strong support for the use of cryptosets to publicly compute of the overlap between private datasets.

## Materials and Methods

### Data for Empirical Studies

**Fig 2:**
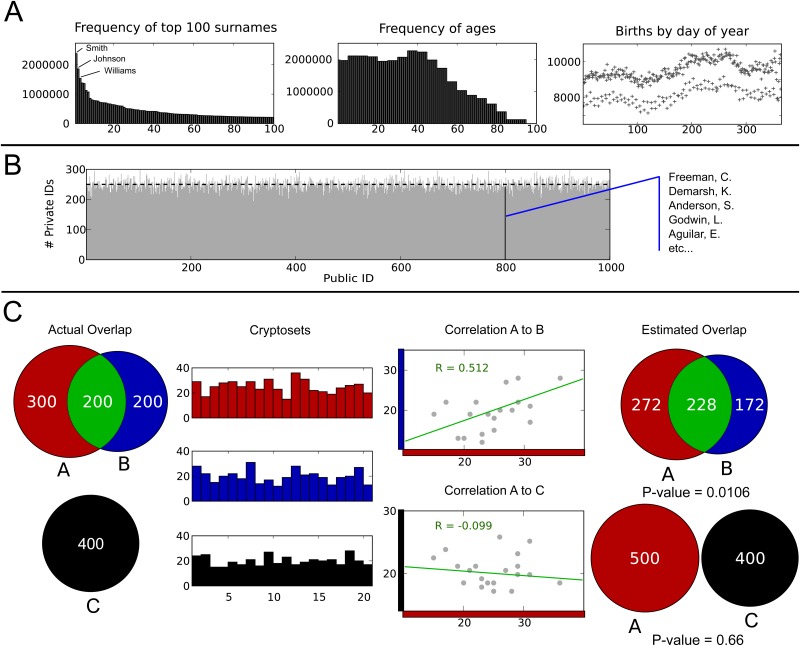
Cryptosets exploit a statistical property of hash functions: they distribute private IDs uniformly across public IDs, assigning several private IDs to the same public ID when truncated. Names and birth dates are distributed with discernible patterns in 2010 census data (Panel A). For example, surnames follow a power law distribution, with a few very common names and a long tail of uncommon names. Birth years fall in a comparatively narrow range. Birth days follow a seasonal and weekly cycle. Nonetheless, hashing 250,000 private IDs—of names with dates of birth—generated from these distributions yields uniformly distributed public IDs (Panel B). Moreover, people binned to the same public ID have no real-world connection to one another. Histograms of these public IDs, cryptosets, can be used to estimate the overlap between private datasets (Panel C). These estimates are computed by multiplying the correlation between cryptosets by the geometric mean of their size. P-values, confidence intervals, and other statistical measures of these estimates can be computed. In the figure, an unrealistically small length of 20 is used for clarity.

**Patient identifiers.** We simulated a population of 250,000 patients to use in simulation studies. A name and a birthday were drawn a random according to the distribution of names and birthdays in US census data (Fig. 2A and 2B). The private ID is defined as the lowercase last name concatenated with the numerical birthdate in mmddyyyy format. Public IDs are defined as the SHA256 hash of the private IDs (concatenated with a salt string) modulo the length of the cryptoset, which varies by experiment. The salt string was used like a random seed by changing it between trials. In practice, the salt string would be fixed and publicly specified.

**Chemical structures.** The aim with this dataset is to estimate the number of scaffolds in common between two chemical libraries. We downloaded two large chemical libraries from PubChem [34]. Molecules were stripped of their side-chains to generate a list of scaffolds in each library [35]. The first library had 49982 scaffolds, the second had 12356 scaffolds, and the two libraries shared 4847 scaffolds in common. Private IDs were defined as the OpenBabel-computed canonical SMILES strings of the scaffolds [36]. Public IDs are defined as the SHA256 hash of the private IDs modulo 100.

### Cryptosets

In this section, the theoretical basis for cryptosets is presented. Cryptoset are first defined. Second, a proof is presented for a formula for computing dataset overlap using cryptosets. Third, an estimate of this estimate’s accuracy is derived form a theoretical framework. Fourth and finally, we derive formulas for computing the information content of cryptosets. The fact that the information gain approaches zero as dataset size grows, while the accuracy states constant, is the key result of this study, demonstrating that cryptosets are information theoretic secure. As we will see, key formulae and theory from this section are verified by experimental studies in the Results section.

**Cryptoset construction.** A cryptoset is defined by two components, both of which are safe to share without compromising security.

1. An identifier regularization protocol that ensures that everyone constructs the same private ID for items to be added to a cryptoset. Clashes in the private ID should be minimized but do not need to be entirely eliminated.
2. A cryptographic hash function *H*(⋅) that maps strings uniformly to an integer space of public IDs ranging from 0 to *L*−1, where *L* is called the “length” of the cryptoset. *L* is typically chosen between 500 to 10000, but could be any positive integer. The smaller the length, the more secure the data-structure. The larger the length, the more accurate the overlap estimates.

From this definition, the cryptoset is computed from a private dataset with the following algorithm. First, compute the regularized, private identifiers for all items in the set. Second, initialize an array 𝒜 of zeros of length *L*. Third, for each identifier *s*, compute its hash *i* = *H*(*s*) to use the public ID for this item. Finally, increment item *A*_*i*_ of 𝒜. The resulting histogram of IDs is the cryptoset which we denoted with a calligraphic letter $𝒜={\left(A_{i}\right)}_{0}^{L-1}$.

**Estimating overlap with cryptosets.** Consider two private datasets, from which cryptosets, 𝒜 and ℬ, of length *L*, with *A* = ∑*A*_*i*_ and *B* = ∑*B*_*i*_ members each are constructed using the same protocol, where *i* ranges from 0 to *L*−1 and the subscripted letters are the number of private IDs for each public ID *i*. We assume that the hash function maps private IDs to public IDs in a manner indistinguishable from a uniform, random process, so the expected counts for elements in each cryptoset are *A*/*L* and *B*/*L*, respectively. Now, there are three types of counts for every item of the datasets. First, there are counts derived from items only in the first dataset, or *A* ^′^ counts. Second, there are counts derived from items only in the first dataset, or *B* ^′^ counts. Third, there are counts derived from items in both datasets, or *A*∩*B* counts.

From this starting point, we model each element of 𝒜 and ℬ as the sum of two of three independent, random variables. Elements of 𝒜 are modeled as the sum of an *A* ^′^ and *A*∩*B* associated variable. Elements of ℬ are modeled as the sum of a *B* ^′^ and *A*∩*B* associated variable. All three variables are characterized by separate Poisson distributions, with rates *A* ^′^/*L*, *B* ^′^/*L* and *A*∩*B*/*L*. Therefore, each cryptoset’s elements follow a Poisson distribution with rates, respectively, (*A* ^′^+*A*∩*B*)/*L* and (*B* ^′^+*A*∩*B*)/*L*. Consequently, the variances of the cryptosets’ elements are

$$\begin{array}{c} Var\left(𝒜\right)\approx \frac{A^{'}+A\cap B}{L} \end{array}$$ (1)

and

$$\begin{array}{c} Var\left(ℬ\right)\approx \frac{B^{'}+A\cap B}{L}, \end{array}$$ (2)

following from the variance of the Poisson distribution (which equals its rate) and the fact that the variance of the sum of independent variables is the sum of the variables’ variances. From here, we see that the product of the covariance of the two cryptosets and *L* approximates the number of common items. In other words,

$$\begin{array}{c} A\cap B=Cov(𝒜,ℬ)\cdot L+\epsilon . \end{array}$$ (3)

Here, *ϵ* is an error term that has zero mean and non-zero variance. Recognizing that the covariance is the Pearson correlation times $\sqrt{AB}/L$, we can also write

$$\begin{array}{c} A\cap B=R_{𝒜ℬ}\sqrt{AB}+\epsilon , \end{array}$$ (4)

Next, by dividing by min [*A*, *B*] we arrive at the estimate for the “overlap proportion”

$$\begin{array}{c} \frac{A\cap B}{\min[A,B]}=R_{𝒜ℬ}\sqrt{\eta }+\epsilon , \end{array}$$ (5)

where *η* is the defined as the ratio between *A* and *B*

$$\begin{array}{c} \eta =\frac{\max[A,B]}{\min[A,B]}. \end{array}$$ (6)

**Theoretical accuracy of cryptoset overlap estimates.** A well-studied statistical framework underlies overlap estimates, because cryptoset comparisons are closely related to the Pearson correlation [37]. The key result from this framework is that the error of the overlap proportion estimate, *ϵ* in Equation 5, is distributed according to an inverse Fisher transformed normal distribution. The Fisher-transform, atanh, is a variance stabilizing transform of the Pearson correlation, and the inverse Fisher-transform is tanh. So *ϵ* is distributed like

$$\begin{array}{c} \epsilon ∼\left(\text{tanh}\left[N\left(\text{atanh}\left[R_{𝒜ℬ}\right],\frac{1}{L-3}\right)\right]-R_{𝒜ℬ}\right)\sqrt{\eta }, \end{array}$$ (7)

or approximately,

$$\begin{array}{c} \epsilon ∼N\left(0,\;\frac{\eta {\left(1-R_{𝒜ℬ}^{2}\right)}^{2}}{L-3}\right) \end{array}$$ (8)

where *L* is the length of the cryptoset (the number distinct public IDs possible) and N(⋅,⋅) is a normal distribution with specified mean and variance. This formula defines significance values, confidence intervals, and critical values for overlap estimates. For example, the endpoints of the 95% confidence interval of an overlap proportion estimate are given by

$$\begin{array}{c} \text{tanh}\left[\text{atanh}\left[R_{𝒜ℬ}\right]\pm 1.96\sqrt{\frac{1}{L-3}}\right]\sqrt{\eta }, \end{array}$$ (9)

Similarily, we can compute the p-value of an overlap estimate, with respect to the “no overlap” null-hypothesis, as

$$\begin{array}{cc} p\text{-value} & =\Phi \left(-\text{atanh}\left[R_{𝒜ℬ}\right]\sqrt{(L-3)/\eta }\right), \end{array}$$ (10)

where Φ(⋅) is the cumulative distribution function of a standard normal distribution. These results expose two important properties of cryptosets. First, cryptoset accuracy is tunable by varying its length, equivalent to changing the number of possible public IDs. Second, cryptoset accuracy does *not* depend on the magnitude of *A* or *B*. Cryptosets compute overlap at a predetermined resolution, no matter how many items are added or removed from them.

**Information content of cryptosets.** Consider a cryptoset 𝒜 with *A* items generated with a public protocol. Here, we mathematically analyze the extent to which this cryptoset compromises the privacy of the items it contains. An item’s security is compromised if we can show that it is a member of the private dataset. In the context of patients in a genomic study of a disease, for example, identifying a patient in the cryptoset would give us the private information that this patient has this disease. As we will see, cryptoset are non-informative about the membership of specific items.

For this analysis, we invoke a “universe” cryptoset 𝒰 = (*U*_*i*_) with *U* items. The cryptoset includes all the items from which cryptoset 𝒜 could have drawn items. The size of this cryptoset *U* is domain specific and much larger than *A*. For example, we might know that people in a cryptoset are drawn from a specific state, so *U* would be the population of the state. As a consequence of the statistical properties of the hash function, 𝒰 is expected to be nearly uniform, with an approximately equal number of counts for every public ID.

We need to know how useful the information in 𝒜 for identifying members of its private dataset. Like the universe cryptoset, 𝒜 will approach a uniform distribution as more items are added to it. If there is only a few items in 𝒜, however, its distribution might be substantially different than uniform. This gives us information about which items are in the private datset that 𝒜 summarizes. We can quantify, exactly, how much information in bits it conveys by computing the information gain (also known as the Kullback-Leibler divergence) between the cryptoset 𝒜 and 𝒰 [38, 39],

$$\begin{array}{c} I\left(𝒜\;∥\;𝒰\right)=\sum_{i}\frac{A_{i}}{A}\log_{2}\left[\frac{A_{i}}{A}\right]-\frac{A_{i}}{A}\log_{2}\left[\frac{1}{L}\right] \end{array}$$ (11)

$$\begin{array}{c} =\sum_{i}\frac{A_{i}}{A}\log_{2}\left[\frac{A_{i}L}{A}\right] \end{array}$$ (12)

using 0 log 0 = 0 where appropriate and asserting that 𝒰 is uniformly distributed.

Assuming our hash function assigns public IDs uniformly across the cryptoset, we can also compute the expected information gain for cryptosets of a specified length *L* and number of items *A*. Here, we substitute in the probability according to a Poisson distribution of observing a count of *k* in an element of the cryptoset

$$\begin{array}{c} E\left[I(𝒜\;∥\;𝒰)\right]=L\sum_{k=1}^{\infty }P\left(k,\frac{A}{L}\right)\frac{k}{A}\log_{2}\left[\frac{Lk}{A}\right], \end{array}$$ (13)

multiplied by the information gain that observing this count would add, where the summation is over possible counts *k*. Rearranging this formula by substituting in the density function for the Poisson distribution, and reparameterizing *k* → *k*+1 so *k* ranges from zero to infinity, we have

$$\begin{array}{c} E\left[I(𝒜\;∥\;𝒰)\right]=\sum_{k=0}^{\infty }\frac{A^{k}\;e^{-A/L}}{L^{k}\;k!}\log_{2}\left[\frac{L(k+1)}{A}\right]. \end{array}$$ (14)

As we will see in extensive experiments, the actual information gain of a cryptoset is very close to this expectation, and goes to zero as the size of the dataset grows.

### Bloom Filters

In this study, Bloom filters are used as a baseline against which to compare cryptosets. In this section, the basic machinery for Bloom filters is presented. Bloom filters are first defined. Second, a formula from the literature for computing overlaps using Bloom filters is presented. Third and finally, we derive formulas for computing the information content of cryptosets. The information content of Bloom filters also approaches zero as dataset size grow, however, the accuracy also goes to zero. Unfortunately, the tight interrelationship between dataset size, security, and accuracy prevents the choice of a Bloom filter length independent of dataset size. This is a fundamental flaw that precludes their use in the applications we propose. Furthermore, as we will see in the Results section, direct comparisons between Bloom filters and cryptosets show that Bloom filters are always less accurate than cryptosets a fixed security level.

**Bloom filter construction.** A bloom filter is defined by two components, both of which are safe to share without compromising security.

1. An identifier regularization protocol that ensures that everyone constructs the same private ID for items to be added to a Bloom filter. Clashes in the private ID should be minimized but do not need to be entirely eliminated.
2. A set of *k* independent cryptographic hash functions *H*_*i*_(⋅) that mapsstrings uniformly to an integer space of public IDs ranging from 0 to *L*−1, where *L* is called the “length” of the filter. *L* is highly dependent on the number of items in the dat set, and is typically chosen to be large enough to limit the possibility of the filter becoming saturated.

From this definition, the Bloom filter is computed from a private dataset with the following algorithm. First, compute the regularized, private identifiers for all items in the set. Second, initialize an array 𝒜 of zeros of length *L*. Third, for each identifier *s*, compute a set of public IDs using all *k* hashes to use for this item, *h*_*i*_ = *H*_*i*_(*s*), and set item *A*_*h*_*i*__ of 𝒜 to 1. The resulting bit vector of IDs is the Bloom filter which we denoted with a calligraphic letter $𝒜={\left(A_{i}\right)}_{0}^{L-1}$.

This algorithm is very similar to the cryptoset algorithm, but has some key differences. First, Bloom filters use a bit vector instead of a integer vector, only strong a binary flag for each public ID. Second, Bloom filters can use more than one hash function. Third, the optimal length of Bloom filters, unlike cryptosets, depends on the size of the database, and cannot be safely fixed ahead of time.

**Overlap estimates using Bloom filters.** Consider two Bloom filters with an observed number of bits turned on in each filter (*a* and *b*, respectively), a specified number of bits turned on in either fingerprint (*a*∪*b*), and using *k* hash functions. The overlap between the elements represented in the Bloom filters is approximately [24]

$$\begin{array}{c} A\cap B\approx -L/k\left(\log\left[1-\frac{a}{L}\right]+\log\left[1-\frac{b}{L}\right]-\log\left[1-\frac{a\cup b}{L}\right]\right). \end{array}$$ (15)

The wart in this formula is its singularity if *a*, *b*, or *a*∪*b* equals *L*. At these singularities, the overlap estimate becomes uncomputable. This condition is met with either filter or the union of the two filter is completely saturated. As dataset size increases, the probability of saturation increases until it becomes a near certainly. So, the chances of an uncomputable overlap increase as dataset size increases.

**Information content of Bloom filters.** Consider a Bloom filter with *A* items, *a* bits turned on, *k* hash functions and length *L*. As with Cryptosets, we can quantify how much information, in bits, a Bloom filter conveys by computing the information gain between the the filter and a fully saturated Bloom filter containing all the items in our universe [38, 39]. Algebraic manipulation reduces this to

$$\begin{array}{cc} I(𝒜\;∥\;𝒰) & =-\frac{a}{A}\log_{2}\left[\frac{a}{L}\right]-\frac{L-a}{A}\log_{2}\left[1-\frac{a}{L}\right]. \end{array}$$ (16)

This equation is derivable by multiple routes, and is essentially the total entropy of the Bloom filter divided by the number of items in the dataset: the information per item. Closed form estimates of the expected information gain are derivable from here, but not included here because they are not used in this study.

## Results

In the following sections we study the accuracy and security of cryptosets for computing overlap between private datasets. These results include both theoretical analysis and empirical studies to demonstrate that cryptosets can securely compute the overlap between private dataset. Finally, we compare Cryptosets to Bloom filters with respect to both accuracy and information gain.

### Accuracy of Overlap Estimates

A good estimate of the overlap can be computed by measuring the Pearson correlation between elements of a cryptoset, and then multiplying the correlation by the geometric mean of the number of elements in each cryptoset. The derivation of this estimate is in the Methods. It might seem surprising that such a simple formula could yield reasonable estimates of accuracy even as the number of items in a cryptoset grows. Nonetheless, this behavior is exactly what we see in both realistic examples and a large simulation with thousands of overlap estimates.

For the first example (Fig. 2C), we simulated three clinical studies (A, B, and C) with 500, 400, and 400 patients each, and with 200 patients shared between studies A and B and no patients in common between studies A and C. We then generated cryptosets with a length of 1000 for each study, and used these cryptosets to estimate the studies’ overlap. Using 1000 Public IDs, the intersection estimate between A and B was 200.1 on average, with a standard deviation of 11.3. The average estimate between A and C was 0.4, with a standard deviation of 14.0. For display purposes, we used 20 Public IDs, an unrealistically small length.

**Fig 3:**
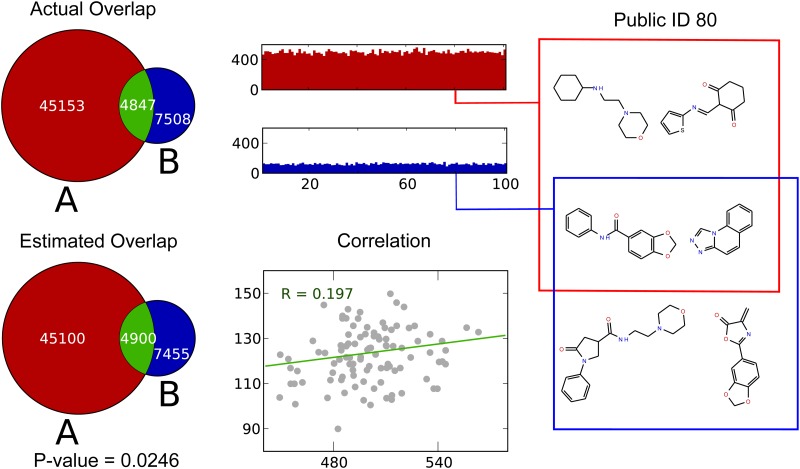
Cryptosets can measure the overlap between chemical collections. In this figure we compare two molecular libraries, which have about 5000 scaffolds in common. The public IDs from the libraries’ scaffolds are nearly evenly distributed across public IDs, but a subtle, statistically significant correlation demonstrates they overlap. The estimated overlap is quite good. Moreover, the privacy of the libraries is maintained. Within each public ID bin (representative examples shown for one bin), there are both scaffolds unique and common to each library, and there is no way to determine which are which from the cryptosets. Sharing overlaps between molecular libraries could help researchers know when it makes sense to screen a private molecule library with a biological assay.

For the second example, we computed the overlap between two chemical libraries (Fig. 3). In this case, there were tens of thousands of scaffolds summarized by cryptosets of just 100 public IDs. Both cryptosets were entirely saturated, looking almost like flat, uniform histograms. Notwithstanding our theoretical results, it intuitively seems like computing overlap would be impossible in this case. Nonetheless, we find the overlap estimates are quite close to the true overlap. This agrees with the theoretical analysis, and demonstrates how our intuition can be misleading on this problem.

**Fig 4:**
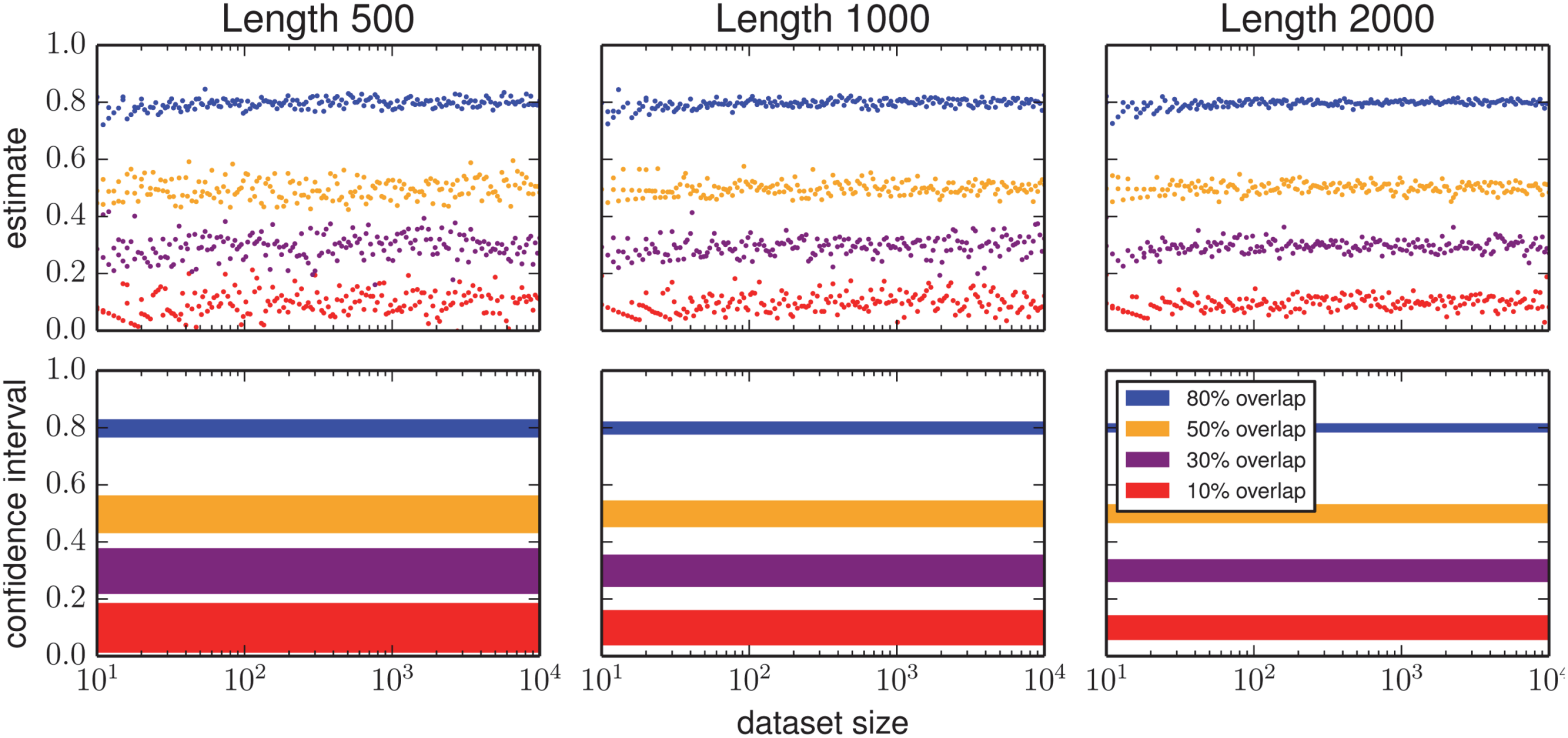
Cryptosets stably estimate the overlap proportion between private datasets, no matter the dataset size, and with accuracy tunable by length. Each column of figures corresponds to a different number of public IDs: 500, 1000 and 2000. The first row shows the results of an empirical study, demonstrating that the error (the spread of each data series) is stable across all dataset sizes. The second row shows the analytically derived 95% confidence intervals (Equation 9), which closely match the distribution of empirical estimates and are stable across all dataset sizes. Also evident in these figures is that estimate accuracy is tuned by the length (the number of possible public IDs) of the cryptosets.

Finally, we systematically studied the overlap estimates across a large range of scenarios. From the patient identifier data, we simulated pairs of private datasets with overlap proportions of 10%, 30%, 50%, and 80%, ranging in size from 10 to 10,000. The correct overlap between each pair was compared to the estimated overlap using cryptosets of length 500, 1000, and 2000 (Fig. 4). We see in this study (1) that the overlap estimate is unbiased, (2) the accuracy of the estimate is exactly proportional to the dataset’s size, meaning the relative error is constant, and (3) 95% confidence intervals quite closely matched the observed results.

Together, these three assessments demonstrate that cryptosets can estimate overlap between private datasets in realistic scenarios. Moreover, the accuracy of the overlap proportion (the relative error) is independent of dataset size. Finally, the theoretical framework we developed can accurately model the distribution of overlap estimates.

### Cryptoset Security

The security of cryptosets in public environments relies on an intentional, many-to-one relationship between private and public IDs. Every public ID maps to hundreds or thousands of possible private IDs. Enumerating the private IDs associated with a specific public ID is difficult, and there is no way to tell which ones correspond with those from the private dataset. Cryptosets, in contrast with other effort in this area, do not rely on the secrecy of the public IDs or the hash function and are resistant to this attack. Rather, their security is a consequence of the statistical properties of hash functions.

**Fig 5:**
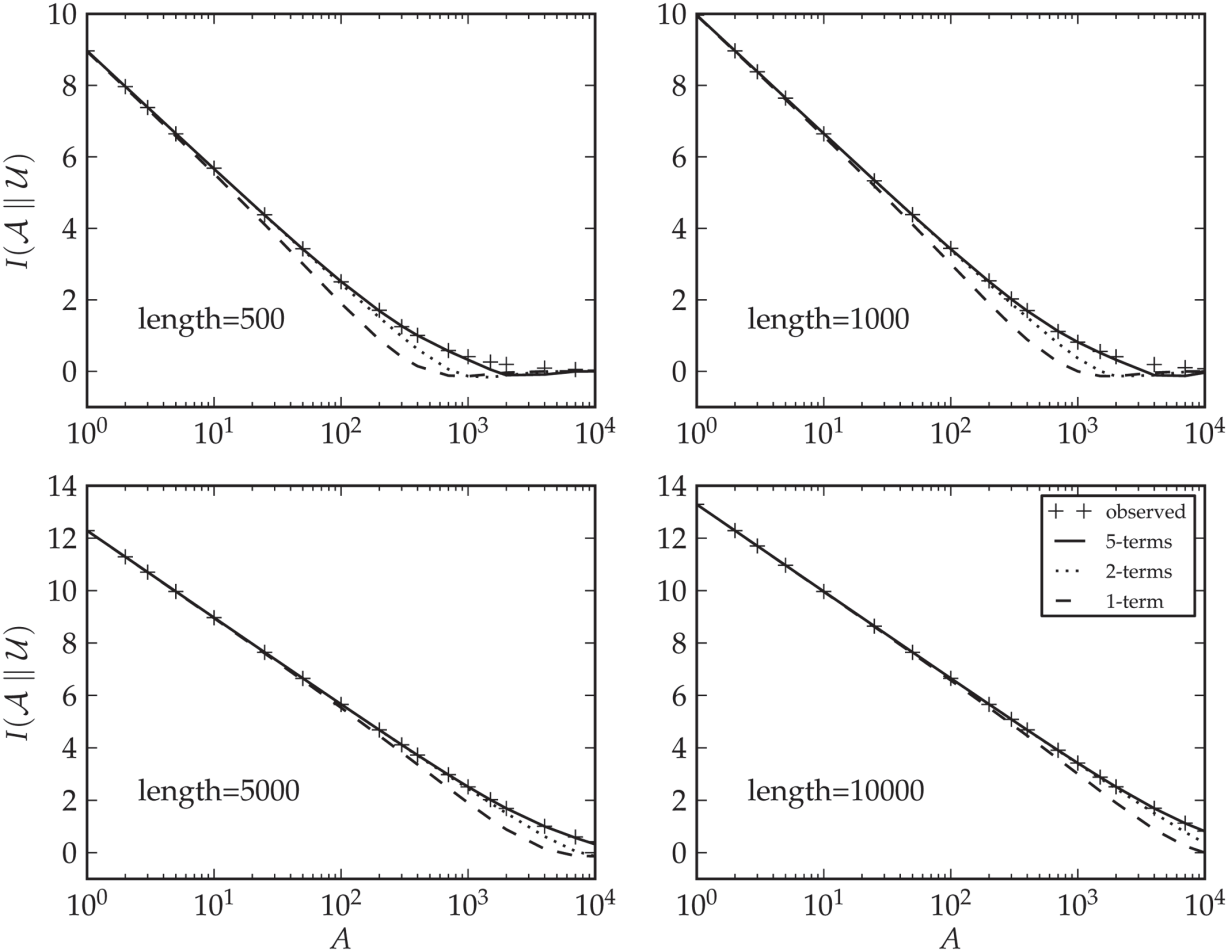
Information gain of cryptosets (y-axis) as a function of number of public IDs (the length) and the number of elements in the cryptoset (A on the x-axis). Our estimates of the information gain are very close to simulations. The curve almost perfectly fits the data with 5 summation terms. A key finding of this study that is evident in this graph is that the information content in cryptosets rapidly approaches zero as the number of items is increased. This makes sense, because as more items are added the distribution of public IDs approaches a uniform, non-informative distribution.

Here, we present an analysis of the security of cryptosets, using information gain as a measure of security risk. Datasets are generated by random sampling, the corresponding cryptosets are constructed for a large range of sample sizes and lengths, and the information gain of these cryptosets is computed (Fig. 5). The key finding from this analysis is that cryptosets become more secure as more items are added. Since the cryptographic hash distributes the private IDs evenly across public IDs (Fig. 2 and 3), the more items in the cryptoset, the closer it is to a uniform distribution and the less informative it is about specific public IDs. As the number of items increases, cryptoset security rapidly approaches “information-theoretic” security, the strongest type of security possible in cryptography, which cannot be broken even with infinite computing power [40–42]. This property makes cryptosets very resistant to attack, even under a malicious model.

### Comparison with Bloom Filters

**Fig 6:**
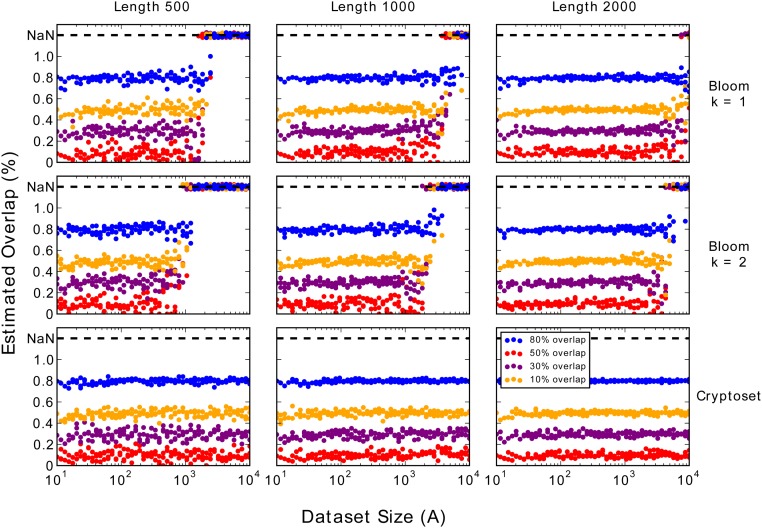
Bloom filter overlap estimates are unstable, with sharp dependence on dataset size. Bloom filters with one (top row) or two (middle row) hash functions can estimate the overlap between two datasets. However, as the dataset size grows along the *x*-axis, the estimate error gradually grows and then the estimates suddenly become uncomputable. Once the dataset size becomes too large, the chance of fully saturating the filters sharply increases, causing a sudden, catastrophic increase in error. At saturation, Bloom filters cannot estimate overlap with any accuracy and are plotted as ‘NaN’ values in the graphs. In contrast, cryptoset accuracy (bottom row) is rock-solid stable, and not dependent on dataset size.

Overlaps between private datasets can be estimated using Bloom filters, but the accuracy of these estimates are unstable with a strong dependence on dataset size (Fig. 6). As dataset size increases, Bloom filter estimates gradually become less accurate, and then suddenly become uncomputable when the datasets are large enough to saturate the filters. The exact dataset size of this transition from computable to uncomputable estimates also depends on the degree of overlap between datasets. Moreover, this transition occurs at smaller dataset sizes when the number of hash functions is increased. In contrast, cryptosets retain the exact same accuracy for all dataset sizes and are never uncomputable.

**Fig 7:**
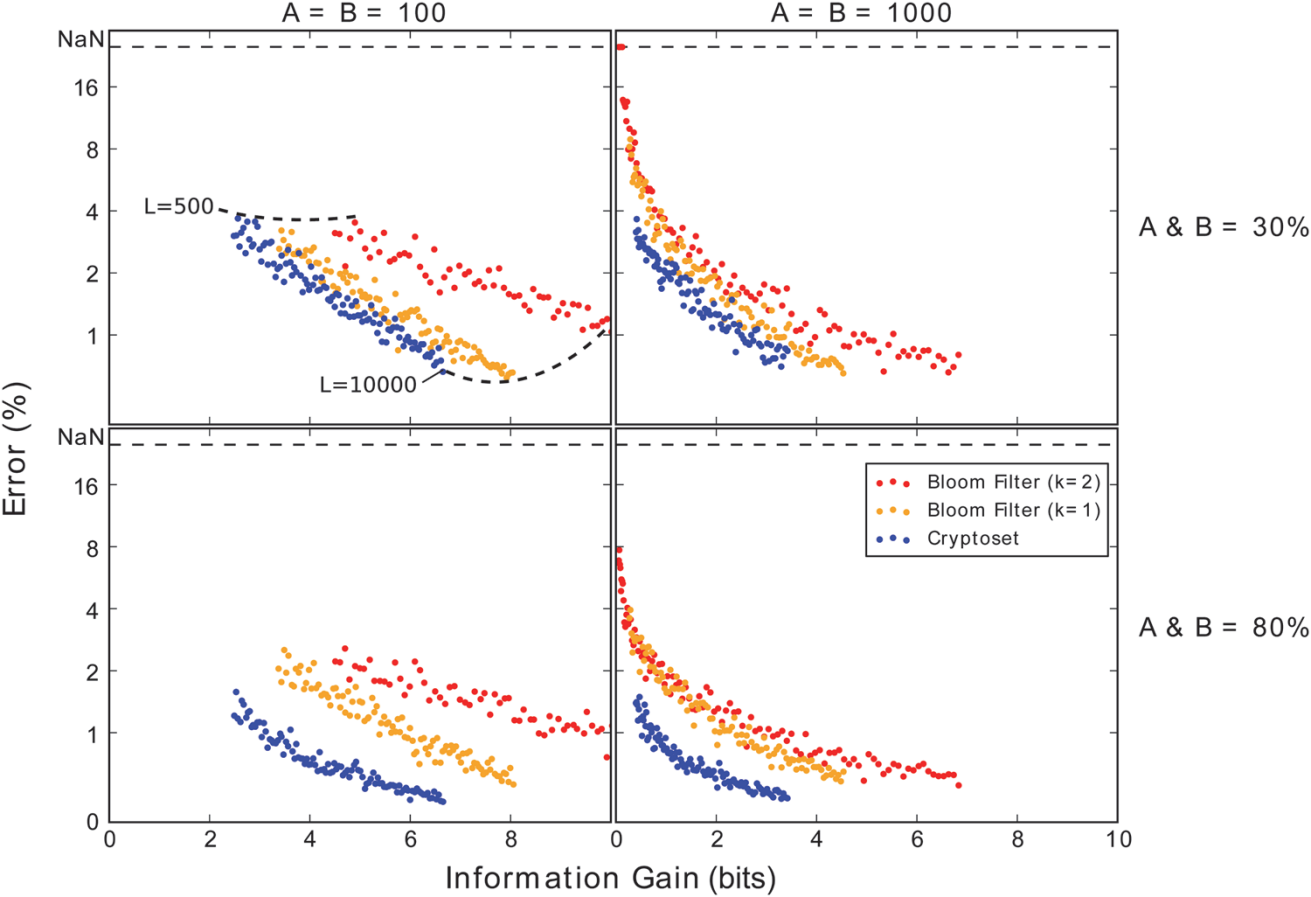
Cryptosets provide a better tradeoff between estimate error and security risk. The figures plot the average over 50 simulations of the overlap estimate error (on a log scale) against average information gain (in bits) for cryptosets and Bloom filters over a large range of lengths (ranging from 500 to 10000). The ideal method would have both error and information gain close to zero. The left column of figures uses datasets with 100 items, while the right column of uses datasets with 1000 items. The top row simulates these datasets with an overlap of 40%, while the bottom row simulates with an overlap of 80%. Cryptosets are consistently more accurate and secure than Bloom filters, and this trend is most pronounced in large datasets with lower overlaps (top right).

There is a trade of between estimate error and security risk. As the number of public IDs increase (the length), estimates become more accurate but the data structure shares more information, becoming less secure. Plotting the trade off between error and information gain (Fig. 7) is another way of comparing Bloom filters and cryptosets. We find that for any given accuracy, cryptosets contain less information than Bloom filters. Moreover, increasing the number of hash functions increases the estimate and the information risk. These results indicate that cryptosets use less information more efficiently than do Bloom filters to make their overlap estimates.

Bloom filters, therefore, are less stable, less accurate, and less secure than cryptosets. As a practical matter, because of the strong interrelationship between filter length, dataset size, and accuracy, it is not possible to pick a single length by which to represent datasets of very different sizes. This complicates the use of Bloom filters, where cryptosets continue to show strong advantages. For example, cryptoset length can be selected without knowing dataset size, based on choosing desired accuracy for overlap estimates. This single length cryptoset will work the same way for the full range of dataset sizes, from very small to very large. Taken together, this set of issues makes cryptosets preferable in our use cases.

## Discussion

We have presented a secure method for computing the overlap between private datasets using cryptosets. Cryptosets appear to be both public and secure, resistant to attack without relying on secret keys or algorithms. At the same time, cryptosets are informative about overlaps between private datasets with a stable, tunable accuracy. Our approach may allow patterns of data sharing that are either impossible or very difficult today. For example, cryptosets of study participants could be published along side genomic studies to enable readers to compute the overlap between, and replication strength of, several studies. A hospital could publish on its website a cryptoset of patients with a rare disease to identify other institutions treating these same patients. A company could publish a cryptoset of its portfolio of compounds to attract collaborators with overlapping lines of research.

Cryptosets are very concise, short enough to be pasted into the body of an email. This conciseness is useful. For example, it is seems intuitively clear that sharing a few hundred integers cannot expose the molecule structures in a large chemical library. Similarly, It also seems plausible that data depositions involving patients (either clinical studies or genomic studies) could include a cryptoset of study participants to facilitate retrospective meta-analysis.

For example, imagine several independent hospitals, outpatient clinics, and pharmacies in the same geographic area. They may want to link some of their medical records together for the purposes of reducing duplicate testing, identifying fraud, detecting drug seeking behavior, or monitoring prescription compliance [43–46]. Sharing the data required to do this on an ongoing basis is difficult, requiring institutional commitment and some unavoidable risk. In this context, only those entities serving overlapping patient populations need to share data. Without directly revealing patient information, the extent to which these populations overlap can be estimated with cryptosets. For example, a hospital might use cryptosets to identify outpatient clinics that serve the patients visiting its emergency room. These clinics could share important health information with the hospital for use in emergencies.

This sharing-privacy dilemma is not unique to health care delivery; it is evident wherever the risks and benefits of sharing are high, and it is particularly acute in genomic medicine. The desire to share the genomic data of large groups of people is only increasing [47–50]. Once patient genomes are routinely sequenced in clinical care, the hope is that genetic data linked to health records can be used to reduce the cost and improve quality of healthcare [51]. However, the sensitive information in health records makes privacy extremely important, so most records are kept private with several barriers to their access. For the foreseeable future, these barriers will remain, rendering research more expensive, difficult and, in some, cases nearly impossible.

Similarily, genomic data is particularly sensitive and important to keep private. First, raw genomic data is essentially impossible to anonymize, especially as sequencing becomes more common [52]. Several studies demonstrate how genetic data can be re-identified and associated with individual patients [53, 54]. For example, individual patients can be re-identified with as few as 75 SNP markers [55]. Even the published SNPs from genome-wide association studies can be used to identify patients [56]. In a particularly important study, recreational genealogy websites were used to link surnames to short tandem repeats in Y chromosomes [57]. Second, a person can never be disassociated from their DNA sequence. Unlike a credit card number, which can be cancelled, a person’s genomic data continues to reveal more private information as our understanding of genetics advances. Third, because DNA sequences are shared between relatives, violating a patient’s genomic privacy also violates the privacy of their relatives.

In situations like this, where both the benefits and risks of sharing data are high, there will probably always remain distinctions between public and private data. With this distinction comes a need to negotiate institutional barriers to sharing.

A key factor that may drive the adoption of this approach is the response of legal departments, institutional review boards, and data repository managers. Cryptosets, with its strong theoretical guarantees, could be treated legally and ethically the same as epidemiological data, like publicly-reported hospital infection rates or monthly number of flu cases. Epidemiological data is review-waived information that can be freely shared because the data does not obvioulsy violate patient privacy. In the same way, cryptosets do not threaten the privacy of individuals. They do enable anyone to compute the overlap between datasets, a very useful function. So it is possible, where this function is important, data repositories could enable and encourage scientists to deposit cryptosets along with their data submissions.

## Conclusion

For the foreseeable future, the conflict between our desire to share data and our need to protect it will continue. Data sharing is protected by many legal and institutional barriers which can require both time and financial resources to successful navigate. However, sharing private data is increasingly necessary for scientific progress in fields that are dominated by sensitive information. Groups assessing the feasible of integrating their data with other organizations want to be guaranteed that the overlap between their private data sets is large enough to validate their efforts. On the other hand, groups looking to expand their private data stores want to be guaranteed of a low overlap so that they are not spending effort acquiring data that they may already have. Cryptosets are an improvement over existing methods, including Bloom filters, and they may be informative and secure enough to help navigate the legal, institutional and financial barriers to accessing data, and enable collaborations and studies that would not otherwise be possible.
